# Hashimoto’s Encephalopathy: A Rare Clinical Diagnosis in a Male Pediatric Patient

**DOI:** 10.7759/cureus.79888

**Published:** 2025-03-01

**Authors:** Mackenzie Herzig, Eduardo Fastag-Guttman, Andrew Wu

**Affiliations:** 1 Emergency Medicine, Hennepin Healthcare, Minneapolis, USA; 2 Internal Medicine, Hennepin Healthcare, Minneapolis, USA; 3 Pediatric Critical Care Medicine, Hennepin Healthcare, Minneapolis, USA

**Keywords:** autoimmune encephalopathy, encephalopathy with autoimmune thyroid disease, hashimoto’s encephalopathy, pediatric critical care usa, pediatric neurology, thyroid disorder

## Abstract

Hashimoto's encephalopathy (HE) is very uncommon in the pediatric literature. It is most often characterized by altered mental status (AMS) that can range from mild confusion to hallucinations and severe agitation. The exact pathophysiologic mechanism of HE is unclear. The most popular proposed mechanism describes an immune-mediated process as opposed to a direct effect of thyroid hormone or thyroid antibodies on the central nervous system (CNS) despite the association in the name with Hashimoto’s thyroiditis. Suspicion for HE is raised when a patient has encephalopathy, the presence of thyroid antibodies, and responsiveness to high-dose corticosteroids.

## Introduction

Hashimoto’s thyroiditis (HT) is the most common cause of hypothyroidism in the United States. It is often suspected when a patient presents with a constellation of symptoms including fatigue, constipation, muscle weakness, depressed mood, memory issues, and dry skin. These symptoms are related to the destruction of thyroid tissue as a result of autoantibody-mediated inflammation of the thyroid, which eventually leads to loss of intrinsic thyroid hormone production [1]. Hashimoto’s thyroiditis occurs more often in the female population, with a male-to-female ratio of 1:10 [1]. A rare complication of HT is encephalopathy, which presents similarly to other types of autoimmune encephalitides. It has only been described in the literature more recently with increasing awareness of the condition since the early 2000s, though there is a case report dating back to 1966 [2]. Hashimoto's encephalopathy (HE) can have various clinical presentations, which makes diagnosis difficult. Most commonly, patients have refractory seizure-like movements, headaches, impaired cognitive function, confusion, behavioral and mood disturbances, disturbance of consciousness, and ataxia. The onset of these symptoms can be insidious or acute. Diagnosis is based on clinical assessment of a patient’s symptoms, positive thyroid autoantibodies in the serum as well as cerebral spinal fluid (CSF), and exclusion of other causes of altered mental status (AMS). Treatment often involves the initiation of high-dose corticosteroids and thyroid hormone replacement [3]. In contrast to the majority of HE reports that characterize the details of the pathology and disease course, this case report is intended to highlight a case of HE in a male pediatric patient, an unusual presentation of an unusual disease.

## Case presentation

A 16-year-old previously healthy male patient was transferred from an outside hospital due to agitation and AMS. Earlier that day he was in his normal state of health, aside from a self-resolved episode of dizziness. Later that evening, a few minutes after a normal conversation, his family heard a thud and found the patient gasping for air with seizure-like activity. When paramedics arrived, the patient was awake but disoriented. En route, he grew more agitated, was hallucinating, and tried to escape the ambulance multiple times, prompting the administration of several doses of droperidol. On arrival, his vitals were normal besides tachycardia of 120 beats per minute. His initial laboratory studies were all normal, including venous blood gas, basic metabolic panel, liver function test, urine drug screen, carbon monoxide, ethanol, acetaminophen, and aspirin levels. Ultimately, the patient was intubated for airway protection due to the onset of emesis amid persistent agitation. The patient was then transferred to our hospital for ongoing care in our pediatric intensive care unit (PICU).

On admission, the patient’s neurological examination was unrevealing. He was encephalopathic, had reactive pupils, withdrew to pain in all extremities, and demonstrated non-sustained clonus in both lower extremities. A computed tomography (CT) scan without contrast of his head was normal. He was also found to be febrile on admission, prompting concern for meningitis, encephalitis, or serotonin syndrome. Lumbar puncture was unsuccessful at bedside, so the patient was empirically treated with meningitic dosing of vancomycin, ceftriaxone, and acyclovir. A midazolam infusion was initiated for sedation, given concern for serotonin syndrome and potential seizure activity. Pediatric neurology was consulted and recommended initiation of levetiracetam and placement of an electroencephalogram (EEG) for seizure evaluation.

**Figure 1:**
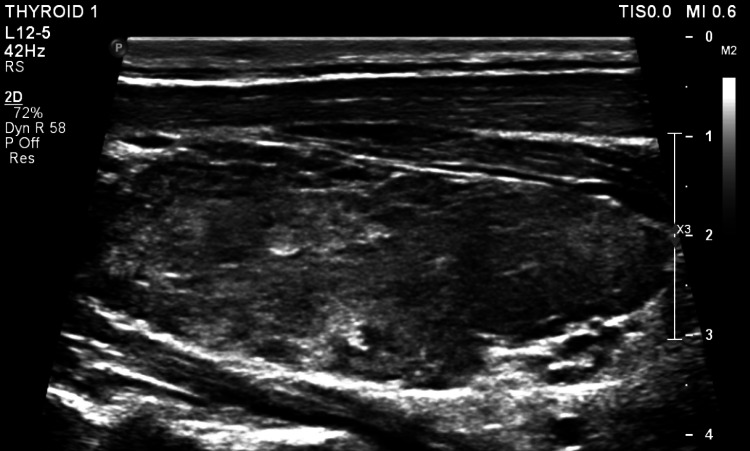
Patient's thyroid ultrasound shows thyroid tissue with hyperechoic nodules of various sizes consistent with Hashimoto's thyroiditis.

**Figure 2:**
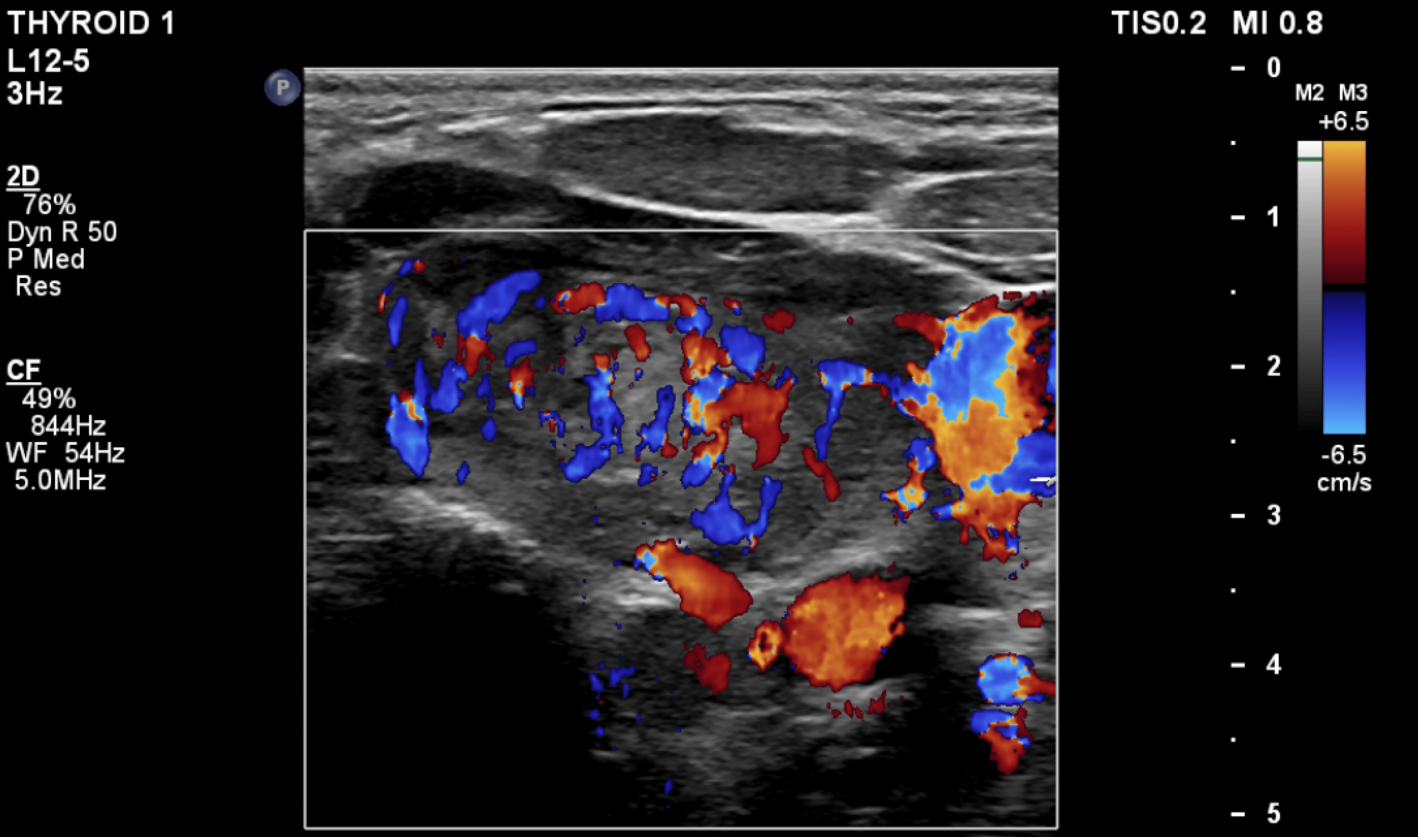
The patient's thyroid ultrasound with color Doppler shows increased blood flow consistent with thyroiditis.

The following day, a lumbar puncture was obtained by interventional radiology, and a magnetic resonance imaging (MRI) of the brain showed no abnormalities. Cerebrospinal fluid studies were unremarkable, including a meningitis/encephalitis polymerase chain reaction (PCR) panel. Additional laboratory evaluations included erythrocyte sedimentation rate, C-reactive protein, procalcitonin, ammonia, blood cultures, toxic alcohol levels, HIV antigen/antibody, urine gonorrhea/chlamydia PCR, and syphilis (rapid plasma reagin (RPR)), all of which were normal or negative. Antibiotics were discontinued after 48 hours of negative blood and CSF cultures. The respiratory viral panel demonstrated the presence of rhinovirus/enterovirus. Given the persistence of AMS, the pediatric infectious disease team was consulted and recommended additional CSF studies, including an autoimmune encephalitis panel, arbovirus antibodies, and Lyme antibodies. Given the lack of definitive findings thus far, thyroid studies were obtained to evaluate for an underlying thyroid disorder responsible for the neurological changes or an arrhythmia that precipitated the initial event. Thyroid-stimulating hormone (TSH) was found to be elevated with low free thyroxine (T4) and total triiodothyronine (T3), as seen in Table 1. A subsequent thyroid ultrasound showed heterogeneous thyroid tissue with increased blood flow and multiple homogeneous hyperechoic nodules in the thyroid, which strongly suggested HT (Figure 1 and Figure 2). Pediatric endocrinology was consulted and recommended measuring thyroid antibodies, including antithyroid peroxidase (anti-TPO), antithyroglobulin antibodies, thyroid stimulating immunoglobulin (TSI), and initiation of levothyroxine.

**Table 1:** Thyroid hormone lab values show the patient’s thyroid hormone levels and thyroid hormone antibody levels.

Laboratory parameters	Value	Reference range
Thyroid-stimulating hormone (TSH)	40 mlU/L	0.27-4.20 mIU/L
Free thyroxine (T4)	0.7 ng/dL	0.9-1.7 ng/dL
Total triiodothyronine (T3)	86 ng/dL	91-218 ng/dL
Antithyroglobulin antibody	1,169 IU/mL	0.0-4.0 IU/mL
Antithyroid peroxidase (anti-TPO)	6,325 IU/mL	0.0-9.0 IU/mL
Thyroid stimulating immunoglobulin (TSI)	34.6 IU/L	< 0.54 IU/L

Numerous attempts were made to wean sedation and work towards a trial of extubation. However, when sedation was weaned, the patient became acutely agitated and would not follow commands. A trial of dexmedetomidine was attempted without improvement and eventually stopped due to the precipitation of bradycardia. Presumed ICU delirium was treated with a trial of antipsychotic medication, which was ineffective. The EEG showed no signs of seizure-like activity, so levetiracetam was discontinued, and the EEG remained negative after discontinuation.

With an otherwise negative workup for the etiology of the patient’s encephalopathy and his new diagnosis of HT, a known autoimmune process, there was a concern for possible underlying autoimmune encephalopathy. Therefore, the patient underwent a three-day course of high-dose methylprednisolone for management of his encephalopathy, hypothesized to be autoimmune in origin. The day after the completion of steroids, the patient was more responsive and alert, and he was successfully extubated. Thyroid antibodies returned at this time, and his antithyroglobulin antibody, anti-TPO, and TSI were all markedly elevated, as seen in Table 1.

After extubation, the patient was transferred from the PICU to the pediatric medical floor and discharged home on hospital day nine with his family after returning to his baseline neurologic status. He was scheduled for outpatient follow-up with pediatric endocrinology and has remained asymptomatic.

## Discussion

Hashimoto’s encephalopathy is an entity that is not well known and is therefore at risk of being underdiagnosed. A patient presenting with AMS prompts a litany of possible diagnoses that are generally more common, leaving HE to be considered later in a patient’s clinical course, and often as a diagnosis of exclusion, after patients have potentially accrued morbidity from the hospital stay. Given its association with HT, HE should be more strongly considered in a patient with HT who presents with sudden onset of a neuropsychiatric symptom. The incidence of HE is even less common in the pediatric population, with less than 80 case reports in the pediatric literature in the past 20 years [4, 5]. Furthermore, HT is approximately 10 times more common in the female population, making our case in a pediatric male patient even more notable [1, 4].

In our young male patient, suspicion for a thyroid-related etiology for his AMS was raised when his work-up in the first 48 hours had been largely unrevealing and he was clinically unchanged. The team discussed all possibilities and systems for potential diagnoses, including thyrotoxicosis and a myxedema coma-like picture, given that both can present with AMS. Our case highlights the importance of considering all physiologic systems when approaching a puzzling case.

The underlying pathology of HE is not well understood. There are a few proposed mechanisms that are noted in the literature, including vasculitis-like processes and other inflammatory processes. Despite the characteristic elevated autoantibodies in HE, there is still a lack of understanding of how the autoantibodies affect the CNS and whether it is from a direct or indirect effect of these antibodies [5]. Two clinical patterns of presentation have been recognized. One primarily manifests similar to that of a stroke with focal neurological symptoms. The other pattern presents with progressive cognitive decline with elements of psychosis. Both presentations can include seizures, which are common in HE [6].

Diagnosis of HE depends on elevated thyroid antibodies, including antithyroglobulin or antithyroid peroxidase. In a review of patients with HE, 48% had elevated antithyroglobulin antibodies and 100% had anti-TPO antibodies, though the level of antibody titers did not seem to correlate with the severity of the disease [6]. Patients with HE can have variable thyroid studies, though more commonly have thyroid hormone levels consistent with euthyroid (50%) or hypothyroidism (47%) [5, 7]. Obtaining an MRI of the brain is recommended as part of the differential workup; however, approximately 50% of patients have a normal MRI, which is consistent with our patient [8].

Hashimoto’s encephalopathy has also been referenced as “steroid-responsive encephalopathy” as most patients respond well to high-dose steroids. There are no current guidelines for steroid dosing for HE, but case reports have shown success with both oral prednisone 50-150 mg daily for four weeks and high-dose intravenous methylprednisolone for three to five days. Some patients have an incomplete response to steroids or require prolonged courses. Other case reports have documented responses to intravenous immunoglobulin, plasma exchange, and rituximab, which are established treatments for other autoantibody-mediated encephalopathies [5, 6, 9]. Once HE is considered as a potential diagnosis, early initiation of steroids is crucial, as most patients have significant improvement in their neurologic symptoms, and earlier treatment portends a more complete and faster recovery. Most patients have complete recovery, but some patients do suffer sequelae, such as cognitive impairment or motor disorders, that vary with residual deficits. On review of cases, at one-year follow-up over 90% of patients had complete resolution of their symptoms; however, about 15% had an episode of recurrence. Recurrence was most common in patients who initially presented with coma [5, 6, 10, 11].

## Conclusions

Hashimoto’s encephalopathy is an uncommon etiology of encephalopathy with limited case reports documenting it, especially in the pediatric population. Due to its rarity, it is often diagnosed later in a patient’s clinical course, potentially after morbidity has been suffered. Patients with HE normally respond well to high-dose steroids, and most return to their baseline neurological status after treatment. Early recognition and treatment of HE are associated with a higher likelihood of complete resolution of symptoms without residual neurologic sequelae. Hashimoto’s encephalopathy is uncommon in the pediatric population and is more commonly diagnosed in the older female population. However, our case demonstrates an unusual case of HE in a pediatric male patient. With the rarity of HE, it is important to keep it on your differential for AMS, especially after you have already ruled out more common causes of AMS and in patients who have a history of Hashimoto’s or other autoimmune conditions. Once considering a diagnosis of HE, prompt initiation of high-dose steroids is crucial for appropriate treatment.
